# Association Between the Nutritional Inflammatory Index and Obstructive Sleep Apnea Risk: Insights from the NHANES 2015–2020 and Mendelian Randomization Analyses

**DOI:** 10.3390/healthcare13070783

**Published:** 2025-04-01

**Authors:** Meixiu Lin, Kaiweisa Abuduxukuer, Lisong Ye, Hao Zhang, Xin Zhang, Shuangshuang Shi, Yan Wang, Yuehua Liu

**Affiliations:** 1 Department of Orthodontics, Shanghai Stomatological Hospital & School of Stomatology, Fudan University, Shanghai 200001, China; 23211370001@m.fudan.edu.cn (M.L.); lsye23@m.fudan.edu.cn (L.Y.); 23111370010@m.fudan.edu.cn (X.Z.); 22211370001@m.fudan.edu.cn (S.S.); 2 Shanghai Key Laboratory of Craniomaxillofacial Development and Diseases, Shanghai 200001, China; haozhang_99@fudan.edu.cn; 3 Department of Preventive Dentistry, Shanghai Stomatological Hospital & School of Stomatology, Fudan University, Shanghai 200001, China; 22211020145@m.fudan.edu.cn; 4 Department of Biostatistics, School of Public Health, Fudan University, Shanghai 200032, China

**Keywords:** obstructive sleep apnea syndrome (OSA), mendelian randomization (MR), disease management

## Abstract

**Background/Objectives:** Current approaches to monitoring obstructive sleep apnea (OSA) risk primarily focus on structural or functional abnormalities, often neglecting systemic metabolic and physiological factors. Resource-intensive methods, such as polysomnography (PSG), limit their routine applicability. This study aimed to evaluate composite nutritional-inflammatory indices derived from routine blood markers to identify feasible indices for OSA management and explore their association with OSA risk. **Methods:** Data from 9622 adults in the NHANES (2015–2020) and GWAS datasets were analyzed using logistic regression, restricted cubic splines, machine learning, and Mendelian randomization (MR). These techniques were employed to identify nutritional-inflammatory indices associated with OSA risk. Random forest modeling identified body mass index (BMI) and albumin (ALB) as key components of the advanced lung cancer inflammation index (ALI). Causal relationships between ALI components and OSA were validated using MR. **Results:** ALI was significantly associated with OSA, with individuals in the highest ALI tertile exhibiting a 59% higher likelihood of OSA (OR = 1.59, 95% CI: 1.38–1.84; *p* < 0.001). BMI and ALB were identified as key contributors to ALI and confirmed as causal risk factors for OSA (BMI: OR = 1.91, 95% CI: 1.80–2.02; ALB: OR = 1.11, 95% CI: 1.04–1.19). Age, gender, and the neutrophil-to-lymphocyte ratio (NLR) were also significant predictors. **Conclusions:** This study identifies ALI as a potential composite index for assessing OSA risk. Integrating statistical modeling, machine learning, and causal inference techniques highlights the utility of nutritional-inflammatory indices in improving OSA monitoring and management in clinical practice.

## 1. Introduction

Obstructive sleep apnea (OSA) is a common respiratory disorder affecting approximately 996 million individuals worldwide, including 425 million with moderate to severe cases [1,2]. It is characterized by recurrent upper airway obstruction during sleep, resulting in apnea, hypopnea, and arousal. OSA is associated with significant complications, such as cardiovascular disease (CVD), metabolic dysregulation, and cognitive impairment, leading to reduced quality of life and shorter lifespan [3,4].

Research has established robust relationships between sleep patterns, inflammatory dietary habits, and nutritional status, highlighting the intricate connections between sleep disorders, nutrition, and systemic inflammation [5,6,7,8,9,10]. Composite nutritional-inflammatory indices—such as the advanced lung cancer inflammation index (ALI), the neutrophil-to-lymphocyte ratio (NLR), the systemic immune-inflammation index (SII), and the prognostic nutritional index (PNI)—have demonstrated utility in predicting conditions like hypertension, CVD, and stroke [11,12]. However, while these combined indices have been extensively explored in other contexts, their application to predicting OSA has been limited.

Among these indices, ALI stands out as a particularly comprehensive marker with the potential to address this gap. Unlike other indices, ALI reflects both systemic inflammation and nutritional status, providing a dual perspective on OSA pathogenesis. It incorporates readily available clinical parameters reflecting both inflammatory and nutritional dimensions of OSA pathophysiology. Specifically, ALI is a composite index that integrates NLR, albumin (ALB), and body mass index (BMI), distinguishing it from previously reported singular markers and offering cost-effective and scalable tools for comprehensive patient assessment. Within this framework, NLR reflects systemic inflammatory status, while ALB and BMI serve as indicators of nutritional state [13,14]. ALI’s successful application in cancer contexts provides a methodological foundation for evaluating nutritional and inflammatory parameters in OSA. Comparative studies demonstrate ALI’s superiority as a nutritional-inflammatory indicator compared to other biomarkers, exhibiting higher area under the curve (AUC) values in predictive models [11,12]. While increased ALI in cancer patients often indicates adequate nutrition and enhanced immunity, in OSA patients, elevated ALI may reflect pathological overnutrition and systemic inflammation. Additionally, epidemiological evidence has established an independent association between OSA and increased lung cancer risk [5]. This epidemiological relationship further supports the potential reverse application of ALI in OSA contexts, where instead of indicating improved health status, the elevated values reflect detrimental overnutrition and inflammatory dysregulation.

Given these advantages, the ALI represents a promising complementary tool for OSA screening, with the potential to enhance early detection, especially in resource-constrained settings. Current diagnostic methods for OSA, such as polysomnography (PSG), portable sleep monitors, and cephalometric analyses, while effective, have significant limitations in routine clinical screening. These methods are resource-intensive, have limited accessibility, and predominantly focus on structural abnormalities rather than addressing underlying systemic pathophysiological factors [15]. In contrast, composite nutritional-inflammatory indices like the ALI provide distinct advantages for OSA screening in primary care settings with readily available clinical parameters from routine medical examinations. They offer a more comprehensive assessment of the multifactorial nature of OSA, integrating both nutritional and inflammatory markers, which is not captured by traditional diagnostic methods.

This study identified ALI as a potential OSA predictor and explored the relationship through multiple analytical methods, including logistic regression, restricted cubic spline analysis, and random forest modeling. Additionally, Mendelian randomization (MR) established causal relationships between ALI component parameters and OSA risk. This research underscoring the importance of integrating nutritional-inflammatory indices into OSA management and demonstrates their potential to enhance diagnostic accuracy, personalize treatment, and improve outcomes. By addressing OSA’s systemic nature, ALI provides a valuable tool for advancing clinical understanding and patient care.

## 2. Materials and Methods

### 2.1. Overall Study Design

This study employed a multi-method approach integrating combined cross-sectional analysis with advanced machine learning and MR techniques. Since all analyses herein were based on publicly available summary data [16], no ethical approval from the ethics committee or institutional review board was required for this study [17,18]. The study adhered to the STROBE guidelines for cross-sectional studies [19,20].

#### 2.1.1. Cross-Sectional Study

- Data Source and Participant Selection

**Figure 1:**
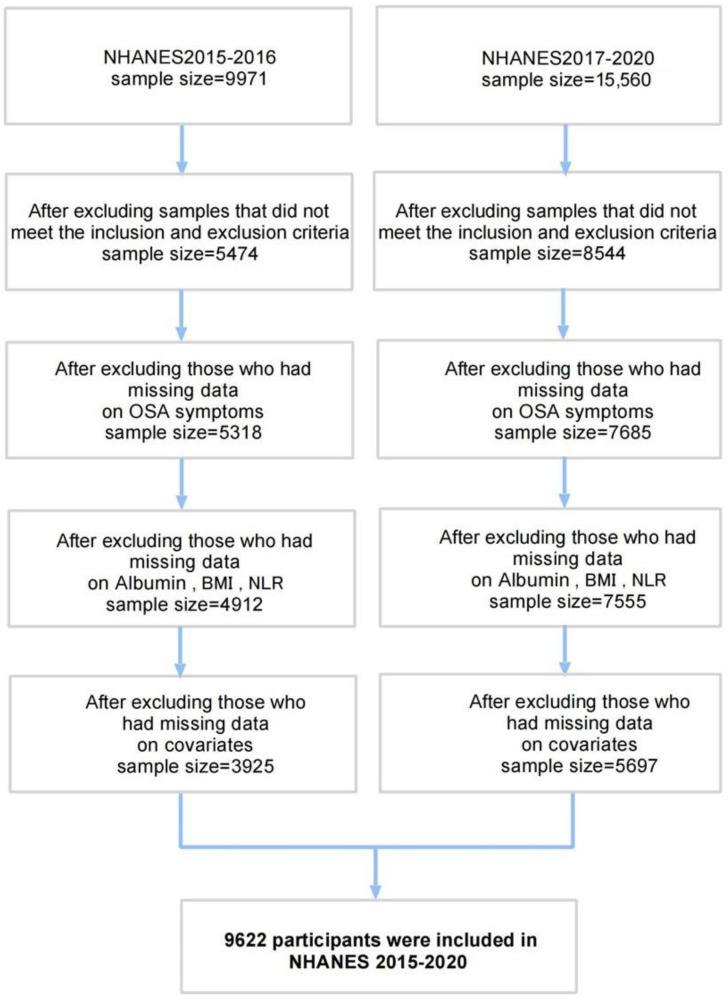
Flow chart of sample selection.

This study used data from the 2015–2016 and the 2017–March 2020 NHANES cycles. After screening an initial pool of 19,225 participants, a total of 9622 individuals aged 20 years or older with complete data on outcome and exposure variables. Patients with severe respiratory dysfunction (a fair amount of wheezing limits usual activity in 12 months; coughing most days over a 3-month period) and cancers were excluded from the study. The detailed participant screening process is illustrated in Figure 1.

- Outcome Definition

Sleep disorders were assessed through questionnaires administered in participants’ homes by trained staff using the Computer-Assisted Personal Interview (CAPI) system [21]. OSA was defined according to the Healthy People 2030 criteria, which include: a doctor-diagnosed sleep apnea; snoring three or more nights per week; snorting, gasping, or stopping breathing three or more nights per week; or feeling excessively sleepy during the day 16–30 times per month despite sleeping seven or more hours per night on weekdays or work nights [22,23].

- Exposure Definition

The calculation formulas for NLR, SII, and PNI are provided in Supplementary S2. ALI was calculated as follows: ALI = BMI (kg/m^2^) × ALB (g/dL)/NLR. Here, BMI refers to body mass index [24]. ALB represents serum albumin levels in grams per deciliter [25,26], and NLR is the neutrophil-to-lymphocyte ratio derived from absolute neutrophil and lymphocyte counts [11,27,28]. ALI was categorized into tertiles: low (≤52.67), intermediate (>52.67 and ≤78.52), and high (>78.52).

- Covariates Definition

Based on prior studies and theoretical considerations, we selected established risk factors for OSA, which are also known to be associated with inflammation and nutritional status, and included them as covariates in our analysis [29,30,31,32,33,34]. We controlled for potential confounding factors through multivariate regression models, adjusting for covariates which included demographic characteristics (age, gender, race, education, marital status), lifestyle behaviors (alcohol consumption, smoking), chronic diseases (diabetes, hypertension, CVD) [35,36], and laboratory data (glycohemoglobin, total cholesterol) [37].

#### 2.1.2. Random Forest Analysis for ALI Components

Random forest analysis was performed using the ‘rfPermute’ package to investigate the association between ALI components and OSA. Variable importance was assessed using Mean Decrease Gini (MDG) and Mean Decrease Accuracy (MDA), identifying key features crucial for OSA risk prediction within the ALI components.

#### 2.1.3. Mendelian Randomization

We performed univariable two-sample Mendelian randomization (MR) analyses to investigate causal relationships between OSA and 233 circulating metabolites published in nature [38], 91 circulating inflammatory proteins, body mass index (BMI), neutrophil count (NC), and lymphocyte count (LC) [6,7,39,40]. We performed linkage disequilibrium (LD) clumping to identify independent single nucleotide polymorphisms (SNPs). We used an r^2^ threshold of <0.001 within a 10 Mb window to ensure that the selected SNPs were independent. Genetic instruments were selected from publicly available GWAS datasets, applying criteria of *p* < 1 × 10⁻^5^, r^2^ < 0.001, and kb < 10,000 for metabolites and inflammatory proteins, and *p* < 5 × 10⁻^8^ for BMI, NC, and LC. The F-statistic, calculated as F = R^2^ (n − 2)/(1 − R^2^), was used to measure the strength of each genetic instrument, where N is the effective sample size for the GWAS of SNP associations, excluding those with F < 10. Outcome data for OSA were obtained from the FinnGen biobank, including 43,901 cases and 366,484 controls, with diagnoses based on ICD codes and confirmed by clinical examinations and sleep studies. Detailed data sources and methods are provided in Supplementary S3, Supplementary S7, and Table S2.

### 2.2. Statistical Analysis

All statistical analyses were performed using R (version 4.4.0) and STATA (version 17.0). For the NHANES cross-sectional study, CDC guidelines were followed by combining multiple survey cycles and applying appropriate sampling weights (Supplementary S4). Baseline differences were assessed using *t*-tests for continuous variables and Chi-square tests for categorical variables. Associations between the four composite indices and OSA were initially explored using univariate logistic regression, followed by multivariate logistic regression for ALI, adjusting for confounders. Effect values are expressed as ORs with corresponding 95% CIs. Restricted cubic splines and two-piecewise linear regression models were used to examine dose–response and non-linear relationships [41]. Subgroup analyses assessed interactions by gender and age. *p*-values < 0.05 were considered statistically significant.

The MR analyses were performed using the TwoSampleMR package and the MR-PRESSO package (version 1.0). MR employed random-effects inverse-variance weighted (IVW) methods to estimate causal effects, controlling for confounding and pleiotropy with MR-Egger and MR-PRESSO tests. Sensitivity analyses, including weighted median and MR-Egger, were conducted to validate findings under different assumptions [39,40,42,43,44].

## 3. Results

### 3.1. General Characteristics of Participants by Tertiles of ALI

The study included 9622 participants, with a mean age of 49 years. The sample was 49.35% female and 36.23% Non-Hispanic White. Educational attainment showed that 58.76% had attended some college or more, 24.91% had never consumed alcohol, and 56.56% had never smoked. OSA was present in 50.44% of participants, with symptoms including snoring (47.58%), snorting or breathing interruptions (12.50%), and excessive sleepiness despite adequate sleep (7.69%). While initially analyzing ALI as a continuous variable demonstrated a statistically significant association with OSA risk, we opted to categorize ALI into tertiles to enhance its clinical interpretability and utility. The population was therefore stratified into three groups based on ALI distribution: low (≤52.67), moderate (52.67–78.52), and high (≥78.52). The prevalence of OSA differed significantly across the ALI tertiles, as shown in Table 1.

### 3.2. Association of Nutritional-Inflammatory Indices with OSA

**Figure 2:**
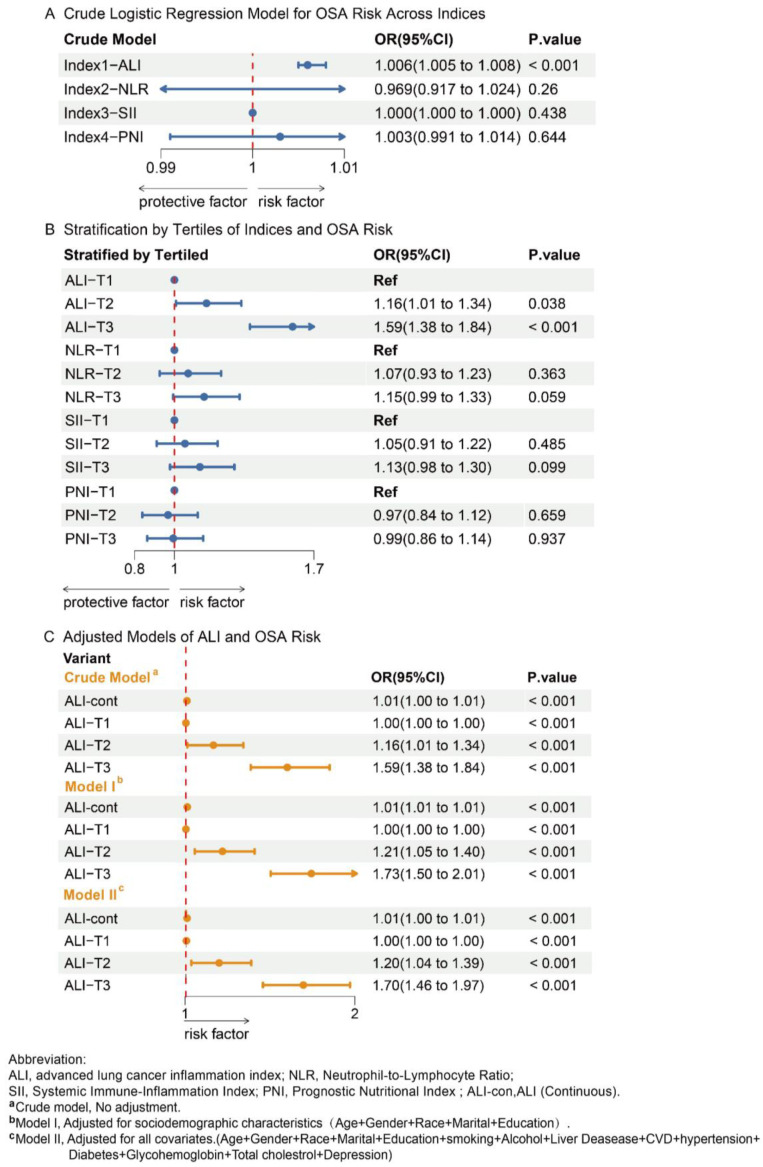
Association of Nutritional Inflammatory Indices with OSA.

Logistic regression models (Figure 2) showed a significant association between ALI and OSA across all models (*p* < 0.001). Stratification by tertiles revealed that OSA risk increased progressively across ALI tertiles (*p* < 0.001), with the highest tertile (T3) exhibiting a notably elevated risk in the crude model (OR: 1.59, 95% CI: 1.38–1.84). In contrast, NLR and other indices (SII and PNI) did not show significant associations with OSA. Figure 2 also highlights that higher ALI tertiles consistently correlated with increased OSA risk, with the highest tertile showing the strongest association (OR: 1.70, 95% CI: 1.46–1.97; *p* < 0.001 in Model II).

Subgroup analysis (Supplementary S5, Figure S1) demonstrated that the relationship between ALI tertiles and OSA risk was consistent across gender and age groups, with higher tertiles generally associated with increased OSA risk. Males and older participants exhibited higher odds ratios in the upper ALI tertiles. However, interaction tests showed no significant differences by gender or age (*p* = 0.212 for gender, *p* = 0.67 for age).

**Figure 3:**
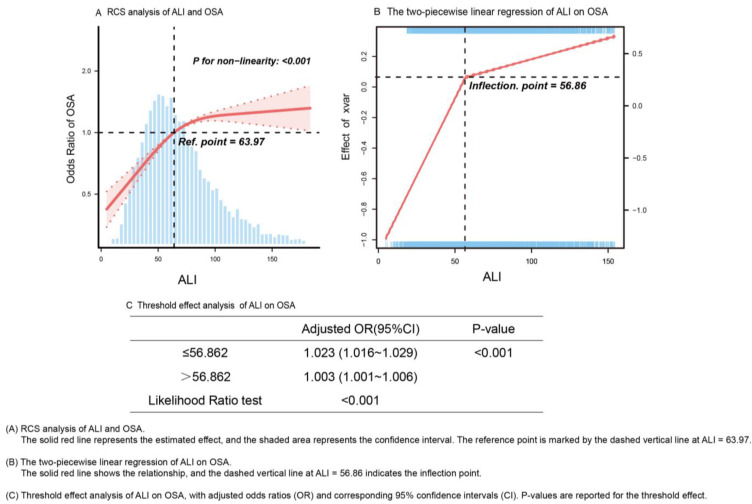
The dose–response relationship and the two-piecewise linear regression between ALI and OSA risks.

Restricted cubic spline analysis (Figure 3) revealed a significant non-linear relationship between ALI and OSA risk (*p* for non-linearity < 0.001). The inflection point at ALI = 56.862 identified through restricted cubic spline analysis holds significant clinical relevance, marking a threshold where the relationship between ALI and OSA risk changes notably. Below this threshold, ALI exhibited a stronger association with OSA risk (adjusted OR = 1.023, 95% CI: 1.016–1.029, *p* < 0.001), with each unit increase in ALI corresponding to a 2.3% higher odds of OSA. Above this threshold, while the association remained statistically significant, its magnitude substantially attenuated (adjusted OR = 1.003, 95% CI: 1.001–1.006, *p* < 0.001). The likelihood ratio test confirmed the statistical significance of this threshold effect (*p* < 0.001). This non-linear relationship suggests that ALI may have particular clinical utility for risk stratification in patients with values below 56.862, where the ALI-OSA association is most pronounced. This identified threshold provides a potential clinically relevant cutoff value for OSA risk assessment and screening prioritization.

### 3.3. Association Between ALI Compositions and OSA

We conducted a sensitivity analysis to assess the association between the components of the ALI and OSA. As shown in Supplementary S6 (Table S1), ALB exhibited an inverse relationship with OSA across all models, while BMI was positively associated with OSA risk. Although NLR was not significant in the crude model, it became inversely significant after adjustment. These findings underscore the individual contributions of ALI components to OSA, reinforcing the significance of ALI as a composite marker for predicting OSA.

### 3.4. Random Forest Analysis of Variable Importance in OSA Prediction

**Figure 4:**
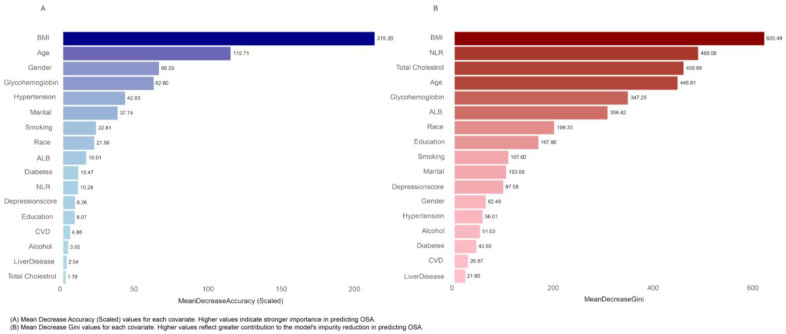
Random Forest Analysis indicating the effects of ALI components and Model II covariates on OSA.

Random forest analysis was used to assess the importance of variables in predicting the association between ALI components and OSA (Figure 4). The analysis showed that BMI had the highest predictive importance, with an MDG of 620.49 and an MDA of 215.26. Other key variables included age, gender, and NLR, each contributing significantly to the prediction model.

### 3.5. MR of ALI Compositions and OSA

**Figure 5:**
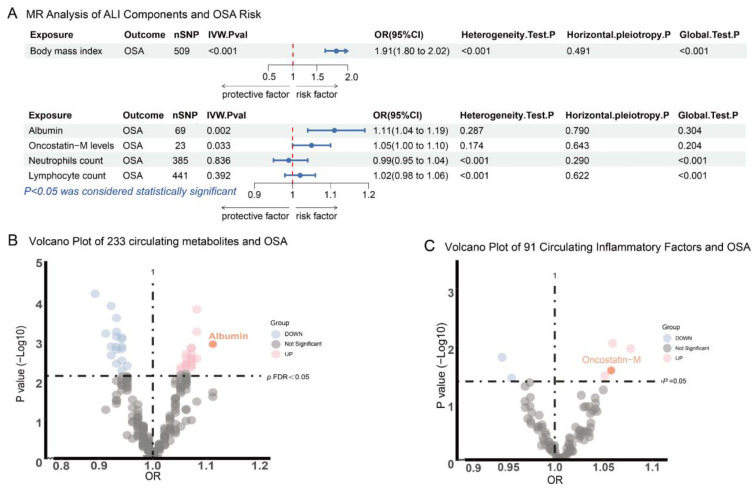
MR analysis of ALI components.

Two-sample MR analyses were conducted to explore the causal relationships between nutritional and inflammatory status and OSA, focusing on ALI components (Figure 5). For nutritional status, we examined the causal relationship between BMI and blood metabolites with OSA. MR analysis revealed a significant causal association between BMI and OSA, with the IVW method yielding an OR of 2.25 (95% CI: 1.73–2.95; *p* = 2.51E-9). No heterogeneity or horizontal pleiotropy was detected.

To further assess metabolic factors associated with OSA, MR was used to investigate causal links between 233 circulating metabolites and OSA (Supplementary S7, Table S3). There are 37 metabolites reached suggestive associations (FDR-adjusted *p* < 0.05) in the IVW analysis. Albumin emerged as a risk factor for OSA (OR = 1.11, 95% CI: 1.04–1.19; *p* = 0.001), with significance maintained after FDR correction (FDR-adjusted *p* < 0.05; Figure 5, Supplementary S7, Table S4). No heterogeneity or horizontal pleiotropy was observed. Metabolites linked to obesity, such as VLDL lipids, also showed suggestive causal links with OSA.

For inflammation, MR was conducted using ALI components—NC neutrophil absolute count and LC lymphocyte absolute count—as exposures, revealing no causal relationship (NC: OR = 0.99, 95% CI: 0.95–1.04; *p* = 0.836; LC: OR = 1.02, 95% CI: 0.98–1.06; *p* = 0.391). Extending the analysis to 91 circulating inflammatory proteins (CIPs), six CIPs showed suggestive associations with OSA before FDR correction (Supplementary S7, Table S3). Notably, the interleukin-6-related factor OSM was linked to an increased risk of OSA (OR = 1.05, 95% CI: 1.00–1.10; *p* = 0.033), but this association was not significant after FDR correction (Supplementary S7, Table S5). Reverse MR analyses were then conducted to investigate potential reverse causality, with OSA as the exposure and BMI, circulating Oncostatin M levels, and albumin as outcomes (Supplementary S7, Table S6). The results indicated that aside from BMI showing a potential role in promoting the development of OSA(OR = 1.14, 95% CI: 1.08–1.19; *p* < 0.001), no causal relationships were observed for the other outcomes (Supplementary S7, Table S7).

## 4. Discussion

This study employs a population-based and genomic approach, integrating statistical models, machine learning, and Mendelian randomization (MR) to evaluate the relationship between a composite nutritional-inflammatory index and OSA. Using NHANES data, inflammatory and nutritional indices, including ALI, NLR, SII, and PNI, were assessed, with ALI showing the strongest association with OSA across age and gender subgroups. Random forest analysis identified BMI, age, gender, and NLR as key predictors. Our study strengthens the established relationship between nutritional status, inflammation, and OSA severity by demonstrating that ALI—which integrates NLR, ALB, and BMI—serves as a comprehensive predictor of OSA risk. These findings underscore the importance of addressing both nutritional and inflammatory factors in OSA management strategies and warrant further validation in diverse populations.

OSA pathogenesis is fundamentally linked to systemic inflammation and metabolic dysregulation, both critical factors in its development and progression. Intermittent hypoxia—a hallmark of OSA—induces both local and systemic inflammatory responses through hypoxia-inducible factor activation and reactive oxygen species generation [45]. Obesity, a major risk factor, contributes to OSA through increased pharyngeal fat deposition, which compromises upper airway patency and enhances collapsibility. Tissue hypoxia caused by upper airway collapse is the main cause of excessive oxidative stress and systemic inflammation in OSA patients [46]. Moreover, visceral adipose tissue functions as an endocrine organ, secreting pro-inflammatory cytokines, including IL-1, IL-6, and TNF-α, activating neutrophils and lymphocytes and promoting chronic low-grade inflammation [8,9]. Importantly, OSA itself exacerbates inflammatory processes through several mechanisms. Sleep fragmentation further disrupts immune regulation by altering oxidative processes, leukocyte function, and cytokine production [47]. Some studies have demonstrated higher oxidative stress, increased levels of reactive oxygen species (ROS), and elevated levels of inflammatory biomarkers in OSA, such as the nuclear factor-κB (NF-κB) [48,49]. The NF-κB family consists of transcription factors that play a critical role as regulators in inflammation. In patients with OSA, the NF-κB pathway is upregulated in adipose tissue. Recurrent episodes of hypoxia-reoxygenation induce oxidative stress and activate NF-κB signaling, resulting in the increased production of inflammatory mediators [46,50,51]. This bidirectional interaction between inflammation and disturbed sleep architecture creates a self-perpetuating cycle connecting inflammation, obesity, and sleep abnormalities.

Our findings align with prior research linking obesity to OSA, as highlighted by Peppard et al. [52], who identified higher BMI as a significant risk factor for OSA, corroborating our MR results (IVW OR = 1.91) [8,52,53,54]. Additionally, we found total cholesterol and obesity-related metabolites, such as very low-density lipoprotein (VLDL), to have suggestive causal links with OSA, underscoring the role of lipid metabolism in OSA pathogenesis. Elevated cholesterol and VLDL lipids, common in obesity, contribute to metabolic disturbances that may exacerbate OSA, highlighting the critical need for effective lipid management [55]. Targeting high cholesterol and VLDL levels through dietary interventions could potentially mitigate OSA severity [56]. Future research should explore the impact of specific dietary patterns and nutritional interventions on OSA, particularly regarding lipid metabolism and systemic inflammation.

Systemic inflammation is a well-established contributor to OSA pathophysiology and severity [57]. OSA is a disease associated with chronic low-grade inflammation. Persistent hypoxia, mechanical airway obstruction, and nocturnal apneas activate the body’s immune response, leading to the onset of inflammation. The chemical structure of albumin can undergo alterations, such as oxidation and glycation, and the modulation of inflammatory responses and metabolic processes. Interestingly, while hypoalbuminemia is traditionally linked to chronic inflammation [58], we found higher albumin (ALB) levels to be associated with increased OSA risk (IVW OR = 1.11). Several factors may explain this phenomenon. The first is the oxidative stress decompensation process in the body. While albumin regulates inflammation, excessive oxidative stress results in the production of high levels of albumin that cannot fully mitigate the long-term chronic inflammation caused by intermittent hypoxia, thereby exacerbating oxidative stress and maintaining a prolonged decompensated state that contributes to the development of OSA. Secondly, in some cases, particularly during the chronic compensatory pathological state of intermittent hypoxia in OSA, the body may elevate albumin levels via mechanisms such as the IL-6/STAT3 signaling pathway in an attempt to counteract the persistent pro-inflammatory environment [59,60]. However, the elevated levels of albumin leading to an increased risk of OSA may be due to the generation of by-products during the body’s inflammatory response, causing metabolic dysregulation and compensatory responses in local tissues and worsening disease. For example, high expression of the IL-6/STAT3 pathway or Complement (C5a/C5aR2 axis) leads to the synthesis and secretion of pro-inflammatory factors [tumor necrosis factor-α (TNF-α), interleukin-6 (IL-6)], reactive oxygen species. It promotes the migration of inflammatory cells, such as neutrophils and macrophages, to the site of injury, thereby exacerbating the inflammatory response. This may promote fibroblast proliferation and collagen deposition, thus increasing the risk of tissue fibrosis [61,62]. Intermittent hypoxia in OSA patients may result in excessive inflammation and complement activation, which could impair tissue repair mechanisms, leading to airway obstruction and other pathological changes.

Additionally, the high expression of inflammatory pathways may be associated with the development of metabolic diseases such as obesity and insulin resistance. In the context of chronic hypoxia, cytokine activation modulates adipocyte function, increases fat deposition, and exacerbates inflammation-related metabolic disturbances by enhancing the secretion of pro-inflammatory cytokines, such as IL-6 and TNF-α, from adipose tissue. Under chronic intermittent hypoxia, multiple mechanisms may contribute to the amplification of inflammation, oxidative stress, immune dysregulation, and metabolic abnormalities. These combined processes may make the inflammatory response triggered by chronic intermittent hypoxia more difficult to control despite a high level of albumin, thereby accelerating the progression of diseases such as OSA. Future studies should further explore the molecular pathways involved in the oxidative stress pathological mechanisms of OSA associated with albumin to provide additional evidence for the correlation between albumin levels and OSA risk. The observed differences between the association of ALB with OSA in our cross-sectional study and the MR analysis may be due to several factors. First, cross-sectional studies are susceptible to confounding factors and gene-environment interactions, which can bias the results. Second, the relationship between ALI and OSA is non-linear, as shown in our dose–response curve (Figure 3). Prior studies have also identified non-linear associations between sleep duration and albumin levels using generalized additive models [18]. The simple logistic model may not fully capture these complex relationships, potentially resulting in an OR < 1 (Supplementary S6). In contrast, MR uses genetic variants as instrumental variables to address confounding and reverse causality, providing more reliable evidence that higher ALB levels may causally increase OSA risk. Further research is warranted to explore ALB’s impact on OSA.

NLR emerged as a significant predictor of OSA in random forest analysis, whereas NC and LC showed no significant associations in MR. This discrepancy may arise from several factors. First, NC and LC, as broad markers of inflammation, might not capture the specific immune processes relevant to OSA. Second, the analysis did not differentiate subtypes of neutrophils and lymphocytes, which may have stronger links to OSA risk, potentially reducing sensitivity. Additionally, cytokines and inflammatory mediators secreted by these immune cells, rather than their counts, may play a more crucial role in OSA pathogenesis [63,64,65,66,67]. Prior studies have highlighted systemic inflammation as a key factor in OSA [68,69], with Yi et al. [70] identifying causal relationships between OSA and elevated CRP, IL-6, and TNF-α. We also found a potential association between oncostatin M (OSM), a cytokine in the IL-6 family, and OSA. OSM initiates inflammatory responses mediated by neutrophils and lymphocytes, underscoring the need to refine ALI by incorporating specific inflammatory markers and immune cell subtypes to enhance its predictive accuracy [71].

Despite significant findings, the study has limitations. The cross-sectional design of NHANES data limits causal inference between ALI components and OSA. While MR offers causal insights, its reliability depends on the availability and quality of genetic instruments [72,73]. ALI, as a composite index, may not fully reflect all aspects of nutritional and inflammatory status relevant to OSA. While components like BMI and ALB are linked to specific genetic markers via GWAS, these associations may not represent the holistic relationship between ALI and OSA, complicating MR validation. Self-reported OSA diagnoses also introduce potential recall bias and misclassification errors [74]. However, the use of validated tools such as the Pittsburgh Sleep Quality Index (PSQI)mitigates some of these issues, and the large NHANES sample enhances reliability [75]. Future research should adopt longitudinal designs to establish temporal relationships, improve diagnostic methods like PSG, and utilize more precise inflammatory and nutritional markers.

While this study has limitations, it establishes a foundation for understanding the relationship between nutritional-inflammatory status and OSA. Additionally, exploring the integration of ALI as a warning tool or regulator into clinical workflows, particularly in primary care settings where PSG may not always be accessible, provides an important avenue for future research. ALI emerges as a practical, cost-effective complement to PSG, addressing systemic factors such as inflammation and nutrition, which are often overlooked by existing diagnostic tools [15]. Integrating ALI into primary care settings, particularly for OSA patients on therapy, could provide a practical approach to monitoring disease progression and therapeutic response in real-world clinical settings. This would allow healthcare providers to track fluctuations in inflammation and adjust treatments without relying on specialized PSG testing. Regular ALI data collection through routine blood draws would reduce the need for visits to specialized sleep centers, lowering healthcare costs and minimizing unnecessary interventions. This approach also empowers healthcare providers to make timely treatment adjustments, improving the delivery of care, particularly in resource-limited settings.

Moreover, ALIs enable clinicians to assess the impact of lifestyle changes, such as diet and weight management, on OSA. Research suggests that dietary and lifestyle modifications—such as reducing pro-inflammatory foods and increasing anti-inflammatory foods—can modulate the inflammatory pathways contributing to OSA severity. For example, diets rich in antioxidants, omega-3 fatty acids, and fiber have been shown to reduce systemic inflammation and potentially alleviate OSA symptoms [76]. By using ALI to monitor the effects of these dietary and lifestyle changes, healthcare providers can offer more personalized treatment plans, enabling patients to make adjustments that could reduce inflammation and improve OSA outcomes. Incorporating ALI into routine clinical assessments and exploring its integration with other biomarkers offers a promising strategy for bridging the gap between advanced diagnostics and practical, cost-effective solutions for OSA management.

This study utilizes the NHANES dataset, which predominantly represents the U.S. population. However, assessing the applicability of albumin levels and inflammation (ALI) in different ethnic and clinical cohorts is essential. While the NHANES database includes populations of varying ethnicities and adjusts for these differences through weighting to ensure accuracy, it is important to acknowledge that even within the same racial group, such as European and American Whites, genetic differences still exist. For instance, the genetic difference in allele frequencies between the same populations in Europe and beyond Europe can be substantial (e.g., a reported difference in certain SNPs, such as those related to common pigmentation variants at the OCA2 gene) [77]. Given the potential variations in genetics, environment, and lifestyle, this consideration is crucial. Racial differences in inflammatory profiles and genetic susceptibility may influence the accuracy of ALI in predicting outcomes. Integrating genetic data from diverse populations, particularly through approaches like Mendelian randomization, could offer a certain valuable insights into the generalizability of ALI. Despite these advancements, further validation through longitudinal cohort studies involving various ethnic and clinical groups remains essential. Future research should focus on validating the ALI as a predictive tool for OSA across diverse populations. Understanding how ALI interacts with these factors across diverse populations will enhance its broader applicability and improve its role in predicting OSA, ultimately leading to more tailored and effective clinical interventions.

## 5. Conclusions

This study highlights ALI as a promising composite marker for OSA risk assessment. Integrating statistical modeling, machine learning, and causal inference techniques provides a comprehensive framework for understanding combined index and underscores the potential of incorporating nutritional-inflammatory indices into clinical practice for enhanced OSA monitoring and management.

## Figures and Tables

**Table 1:** Characteristics of participants by Tertiles of ALI.

	Participant Group				
Characteristics	Overall (*N* = 9622)	ALI-T_1_ (*N* = 3211)	ALI-T_2_ (*N* = 3204)	ALI-T_3_ (*N* = 3207)	*p*-Value
Age (years)	49.42 ± 17.32	50.41 ± 0.56	46.71 ± 0.54	45.56 ± 0.45	<0.0001
Gender (%)					0.0299
Male	4874 (50.65%)	1644 (46.78%)	1624 (51.10%)	1606 (50.35%)	
Female	4748 (49.35%)	1567 (53.22%)	1580 (48.90%)	1601 (49.65%)	
Race (%)					<0.0001
Mexican American	1396 (14.51%)	397 (6.80%)	500 (8.78%)	499 (10.27%)	
Other Hispanic	1134 (11.79%)	379 (6.57%)	398 (7.06%)	357 (7.04%)	
Non-Hispanic White	3486 (36.23%)	1467 (72.47%)	1170 (66.89%)	849 (57.22%)	
Non-Hispanic Black	2179 (22.65%)	465 (5.74%)	623 (8.35%)	1091 (16.94%)	
Education (%)					0.32
Less than high school	1760 (18.29%)	616 (11.34%)	578 (9.43%)	566 (10.60%)	
High school	2208 (22.95%)	743 (24.36%)	736 (24.66%)	729 (22.74%)	
Some college	3143 (32.66%)	1013 (31.03%)	1030 (31.25%)	1100 (33.69%)	
Bachelor’s degree or higher	2511 (26.10%)	839 (33.27%)	860 (34.66%)	812 (32.97%)	
Martial status (%)					0.0209
Married/living with partner	5963 (61.97%)	1971 (65.88%)	2032 (67.70%)	1960 (65.71%)	
Widowed/divorced/separated	1757 (18.26%)	640 (16.41%)	556 (14.71%)	561 (13.78%)	
Never married	1778 (18.48%)	558 (16.49%)	569 (16.63%)	651 (19.77%)	
Separated	124 (1.29%)	42 (1.22%)	47 (0.96%)	35 (0.74%)	
OSA (%)					<0.0001
Yes	4853 (50.44%)	1429 (43.56%)	1612 (47.26%)	1812 (55.16%)	
No	4769 (49.56%)	1782 (56.44%)	1592 (52.74%)	1395 (44.84%)	
Albumin (g/dL)	4.18 ± 0.36	4.16 ± 0.01	4.25 ± 0.01	4.26 ± 0.01	
BMI (kg/m^2^)	29.50 ± 6.54	26.84 ± 0.17	29.48 ± 0.17	32.02 ± 0.22	
Smoking (%)					<0.0001
Never	5442 (56.56%)	1698 (54.15%)	1819 (55.39%)	1925 (59.15%)	
Former	2353 (24.45%)	814 (25.02%)	784 (27.30%)	755 (26.51%)	
Current	1827 (18.99%)	699 (20.83%)	601 (17.31%)	527 (14.35%)	
Alcohol (%)					0.117
Yes	7225 (75.09%)	2374 (79.28%)	2425 (82.10%)	2426 (81.06%)	
No	2397 (24.91%)	837 (20.72%)	779 (17.90%)	781 (18.94%)	
Liver disease (%)					0.2135
Yes	473 (4.92%)	147 (3.87%)	172 (5.14%)	154 (4.55%)	
No	9149 (95.08%)	3064 (96.13%)	3032 (94.86%)	3053 (95.45%)	
CVD (%)					<0.0001
Yes	806 (8.38%)	370 (8.69%)	225 (5.32%)	211 (5.57%)	
No	8816 (91.62%)	2841 (91.31%)	2979 (94.68%)	2996 (94.43%)	
Hypertension (%)					0.0244
Yes	3444 (35.79%)	1164 (31.45%)	1077 (29.47%)	1203 (33.07%)	
No	6178 (64.21%)	2047 (68.55%)	2127 (70.53%)	2004 (66.93%)	
Depression (%)					0.0993
No	7202 (74.85%)	2377 (76.16%)	2430 (78.26%)	2395 (74.80%)	
Mild	1619 (16.83%)	561 (15.90%)	529 (15.43%)	529 (17.01%)	
Moderate	507 (5.27%)	159 (5.02%)	165 (4.26%)	183 (5.65%)	
Severe	294 (3.06%)	114 (2.92%)	80 (2.05%)	100 (2.54%)	
Diabetes (%)					0.3291
Yes	1364 (14.18%)	491 (11.52%)	440 (10.29%)	433 (9.84%)	
No	8008 (83.23%)	2648 (86.57%)	2683 (87.61%)	2677 (87.56%)	
Borderline	250 (2.60%)	72 (1.91%)	81 (2.10%)	97 (2.60%)	
Total Cholestrol	4.91 ± 0.03	4.83 ± 0.04	4.92 ± 0.03	4.99 ± 0.03	0.0001
Glycohemoglobin (%)	5.64 ± 0.02	5.61 ± 0.03	5.65 ± 0.02	5.67 ± 0.02	0.0236

Abbreviation: BMI—Body Mass Index (kg/m^2^); CVD—Cardiac Vascular Disease; OSA—Obstructive Sleep Apnea; T_1_—Tertile1; T_2_—Tertile2; T_3_—Tertile3.

## Data Availability

NHANES data are publicly available through the Centers for Disease Control. (https://www.cdc.gov/nchs/nhanes/, accessed on 30 March 2025). GWAS data are available through the MRC IEU Open GWAS database (https://gwas.mrcieu.ac.uk/, accessed on 30 March 2025). The datasets used and/or analyzed during the current study are available from the corresponding author upon reasonable request.

## Supplementary Materials

The following supporting information can be downloaded at: https://www.mdpi.com/article/10.3390/healthcare13070783/s1, S1. Process of Sample Selection. S2. Other Combined indices and OSA risk (Table S1). S3. MR Analysis Data Source. S4. Analyses of NHANES data CDC guidelines. S5. Subgroup analysis of the relationship between ALI tertiles and OSA risk across different gender and age groups (Figure S1). S6. Weighted logistic models evaluating the association between ALI and OSA (Table S2). S7. Mendelian Randomization Analyses results (Refer to Tables S3–S8 in the Mendelian Randomization results file (xls)).

## Abbreviations

ALB	Albumin
ALI	Advanced lung cancer inflammation index
AUC	Area under curve
BMI	Body mass index
CAPI	Computer-assisted personal interview
CIPs	Circulating inflammatory proteins
CIs	Confidence intervals
CVD	Cardiovascular disease
GWAS	Genomic-wide association studies
IL-1	Interleukin-1
IL-6	Interleukin-6
IVW	Inverse variance weighted
LC	Lymphocyte counts
MR	Mendelian randomization
MDA	Mean decrease in accuracy
MDG	Mean decrease Gini index
NC	Neutrophil counts
NF-κB	The Nuclear Factor-κB (NF-κB) family
NHANES	National Health and Nutrition Examination Survey
NLR	Neutrophil-to-lymphocyte ratio
OR	Odds ratio
OSA	Obstructive sleep apnea syndrome
OSM	Oncostatin-M
PLR	Platelet-to-lymphocyte ratio
PNI	Prognostic nutritional index
pQTL	Genome-wide proteome quantitative trait loci
ROS	Reactive oxygen species
SEs	Standard errors
SII	Systemic immune-inflammation index
TNF-α	Tumor Necrosis Factor-alpha
PSQI	Pittsburgh Sleep Quality Index Questionnaire
