# Supplementary material for: Association Between the Nutritional Inflammatory Index and Obstructive Sleep Apnea Risk: Insights from the NHANES 2015–2020 and Mendelian Randomization Analyses
Source: Healthcare (Basel). 2025 Apr 1;13(7):783. doi: 10.3390/healthcare13070783 (PMC11988625; doi:10.3390/healthcare13070783)
Supplement: Supplementary file 1 [file healthcare-13-00783-s001.zip › Supplementary Materials.pdf]

Supplementary Material

1 Supplementary Data

Supplementary S1. Flow Chart of Sample Selection

This flow chart outlines the sample selection process from the NHANES 2015-2020 datasets. The left path represents data from NHANES 2015-2016 (initial sample size = 9971), and the right path represents data from NHANES 2017-2020 (initial sample size = 15560). Participants were excluded sequentially for being younger than 20 years old, missing data on OSA (Obstructive Sleep Apnea) symptoms, missing data on Albumin, BMI (Body Mass Index), NLR (Neutrophil-to-Lymphocyte Ratio), and other covariates. The final included sample size was 9622 participants(Figure1).

Supplementary S2. Other Combined indices and OSA risk(Supplemenatary TableS1).

The formulations of other combined indices：

- Neutrophil-to-Lymphocyte Ratio (NLR):

NLR was calculated by dividing the absolute neutrophil count (NC) by the absolute lymphocyte count (LC). The formula is as follows:

$$\text{NLR} = \frac{\text{Neutrophil Count (NC)}}{\text{Lymphocyte Count (LC)}}$$

- Systemic Immune-Inflammation Index (SII):

The SII was calculated using the following formula, which incorporates platelet count (PLT),

neutrophil count (NC), and lymphocyte count (LC):

$$\text{SII} = \frac{\text{Platelet Count (PLT)} \times \text{Neutrophil Count (NC)}}{\text{Lymphocyte Count (LC)}}$$

- Prognostic Nutritional Index (PNI):

The PNI was calculated using serum albumin levels and total lymphocyte count (TLC), with the following formula:

$$\text{PNI} = (10 \times \text{Serum Albumin Level (g/dL)}$$

$$+0.005 \times \text{Total Lymphocyte Count(TLC)})$$

Supplementary S3. MR Analysis Data Source

We conducted univariable two-sample Mendelian randomization (MR) analyses to examine the causal links of 233 circulating metabolites, 91 circulating inflammatory proteins, BMI, neutrophil count(NC), and lymphocyte count(LC) with OSA[1].In terms of Mendelian randomization analysis, all GWAS summary data used in this study were publicly available in open-access databases. For human blood metabolites, we selected the latest GWAS for 233 circulating metabolic traits published in Nature[2]. This study included 213 lipid and lipoprotein parameters or fatty acids and 20 non-lipid traits quantified by nuclear magnetic resonance spectroscopy in up to 136,016 participants (88.4% European and 11.5% Asian) from 33 cohorts. For circulating inflammatory proteins, we utilized data from a genome-wide proteome quantitative trait loci (pQTL) analysis conducted on 91 circulating inflammatory proteins (CIPs) across 11 cohorts, encompassing a total of 14,824 participants[3]. The summary-level GWAS data for all 91 CIPs are available for download from the EBI GWAS Catalog, with IDs ranging from GCST90274758 to GCST90274848. The criteria for selecting genetic instruments for metabolites and CIPs were as follows: $P < 1 \times 10^{-5}$, $r^2 < 0.001$, and kb < 10,000.

The GWAS data for BMI, comprising 526,508 individuals[4], was acquired from the Genetic Investigation of Anthropometric Traits (GIANT) Consortium. The data for NC (n=519,288) and LC (n=524,923) were extracted from a comprehensive blood phenotype GWAS[5]. The criteria for the genetic instruments for BMI, neutrophil count, and lymphocyte count were as follows: $P < 5 \times 10^{-8}$, $r^2 < 0.001$, and kb < 10,000. The F-statistic, calculated as $F = R^2 (n-2) / (1-R^2)$, was used to measure the strength of each genetic instrument, where N is the effective sample size for the GWAS of SNP associations. Instrumental variables with low F-statistics (<10) were excluded from our analysis. For outcome data, summary-level data for OSA were extracted from a comprehensive European population-wide GWAS conducted by the FinnGen biobank project (www.finngen.fi/en)using the publicly available R10 version. This dataset includes 43,901 OSA cases and 366,484 controls. The diagnosis of OSA was based on International Classification of Diseases codes (ICD-10: G47.3; ICD-9: 3472A) and was confirmed through subjective symptoms, clinical examinations, and sleep registrations (apnea-hypopnea index (AHI) ⩾ 5/hour or respiratory event index (REI) ⩾ 5/hour).

Supplementary S4. Analyses of NHANES data CDC guidelines

The study was conducted according to the Centers for Disease Control and Prevention (CDC) guidelines for the analysis of NHANES data. The data combine multiple cycles and construct appropriate weights according to the NCHS(S(National Health and Nutrition) provided analysts.

Analyses were conducted according to the Centers for Disease Control and Prevention (CDC) guidelines for the analysis of NHANES data. A weights (WTSA2YR), stratum (SDMVSTRA), and primary sampling units (SDMVPSU) were taken into account for the complex survey design. Weighted data were calculated according to analytical guidelines (NHANES:2015 – 2016 and 2017 – 2020). Various sample weights, such as interview weight (wtint2yr), MEC (Mobile Examination Center) exam weight (wtmec2yr), and several subsample weights, are available in NHANES data release file. The selection of the correct sample weights for the analyses depends on the variables used. All the interview and interview weights (wtint2yr) covered in this study are available in the demographic files. Since the sample persons are a subset of those interviewed in the survey, we used the combined interview weight (wtint2yr)for analysis.

The data were weighted according to the information that the NCHS(National Health and Nutrition) provided analysts on how to combine multiple cycles and construct appropriate weights.

NHANES2015-2016 involved a combination of one survey cycles (two years),and NHANES2017-2020 involved a combination of prepandemic special survey cycles (3.25 years). The weights formula of NHANES2015-2016 samples is WTINT2YR = 2/5.25 × WTMEC2YR.

Supplementary S5. Subgroup analysis of the relationship between ALI tertiles and OSA risk across different gender and age groups (Supplementary FigureS1)

The weights formula of NHANES2017-2020 is WTINT3.25YR = 3.25/5.25 × WTMECYR.This figure presents the subgroup analysis of the association between ALI tertiles and OSA risk across different gender and age groups.

Gender: The effect of ALI on OSA risk is shown separately for males and females, with trend tests indicating increasing OSA risk across ALI tertiles. Interaction p-value = 0.212, suggesting no significant difference in the association by gender.

Age: The analysis is stratified by age groups (20–29, 30–39, 40–49, 50–59, 60–69, and 70+ years), showing a consistent trend of increasing OSA risk across ALI tertiles in most age groups. The interaction p-value = 0.67, indicating no significant variation in the association by age.

This figure underscores the consistent association between higher ALI tertiles and increased OSA risk across both gender and age subgroups, without significant interactions by these factors.

Supplementary S6. Weighted logistic models evaluating the association between ALI and OSA. (Refer to Table S2 in the Appendix file (xls))

This table shows the associations between ALI components (albumin, BMI, NLR) and OSA risk across three models

- Crude Model: ALB was inversely associated, BMI positively associated, and NLR not significant.

- Model I: Adjusted for sociodemographic factors; ALB remained inversely associated, BMI remained positive, and NLR became significant.

- Model II: Adjusted for additional covariates; ALB and BMI maintained their associations, and NLR remained significant.

These results highlight ALB as a protective factor and BMI as a risk factor for OSA, consistent across all models.

Supplementary S7. Mendelian Randomization Analyses results （Refer to Tables S3, S4, S5, S6, S7 and S8 in the Appendix file (xls))

Supplementary 7, Table S1. Genetic Instruments for Immune Cells and Inflammatory Proteins Used in Mendelian Randomization Analyses
This table presents the single nucleotide polymorphisms (SNPs) selected as instrumental variables

(IVs) for immune cell traits and circulating inflammatory proteins, including the F-statistics used to assess their strength. All IVs exceeded the F-statistic threshold of 10, indicating a low likelihood of weak instrument bias.

Supplementary S7, Table S3. Metabolites Associated with Obstructive Sleep Apnea (OSA) in Mendelian Randomization Analyses
This table summarizes the causal relationships between 233 circulating metabolites and OSA identified through Mendelian randomization using the inverse-variance weighted (IVW) method. A total of 37 metabolites demonstrated suggestive associations with OSA (FDR-adjusted $P < 0.05$), including those linked to obesity such as VLDL lipids.

Supplementary S7, Table S4. Albumin as a Risk Factor for OSA in Mendelian Randomization Analyses
This table details the association of albumin levels with OSA, showing a significant causal relationship (OR = 1.11, 95% CI: 1.04–1.19; P = 0.001). The association remained significant after false discovery rate (FDR) correction, underscoring albumin's potential role as a risk factor for OSA.

Supplementary S7, Table S5. Inflammatory Proteins Associated with OSA in Mendelian Randomization Analyses
This table explores the association between 91 circulating inflammatory proteins (CIPs) and OSA. Six proteins exhibited suggestive associations before FDR correction, with interleukin-6-related factor OSM showing a potential link to increased OSA risk (OR = 1.05, 95% CI: 1.00–1.10; P = 0.033). However, none of these associations remained significant after FDR correction.

Supplementary S7, Table S6 & S7 . Reverse MR analyses

Reverse MR analyses were conducted to investigate potential reverse causality, with OSA as the exposure and BMI, circulating Oncostatin M levels, and albumin as outcomes.

Supplementary Figures

Supplementary Figure S1. Subgroup analysis of the relationship between ALI tertiles and OSA risk across different gender and age groups

Subgroup	Variable	n.total	n.event_%	P.for.interaction
**Gender**				
Male				**0.212**
	ALI_Tertile1	1644	847 (51.5)	
	ALI_Tertile2	1624	884 (54.4)	
	ALI_Tertile3	1606	957 (59.6)	
	Trend.test	4874	2688 (55.1)	
Female				
	ALI_Tertile1	1567	582 (37.1)	
	ALI_Tertile2	1580	728 (46.1)	
	ALI_Tertile3	1601	855 (53.4)	
	Trend.test	4748	2165 (45.6)	
**Age**				
20−29				**0.67**
	ALI_Tertile1	488	123 (25.2)	
	ALI_Tertile2	557	194 (34.8)	
	ALI_Tertile3	572	223 (39)	
	Trend.test	1617	540 (33.4)	
30−39				
	ALI_Tertile1	458	168 (36.7)	
	ALI_Tertile2	557	261 (46.9)	
	ALI_Tertile3	553	308 (55.7)	
	Trend.test	1568	737 (47)	
40−49				
	ALI_Tertile1	478	235 (49.2)	
	ALI_Tertile2	563	303 (53.8)	
	ALI_Tertile3	580	369 (63.6)	
	Trend.test	1621	907 (56)	
50−59				
	ALI_Tertile1	497	269 (54.1)	
	ALI_Tertile2	552	316 (57.2)	
	ALI_Tertile3	580	366 (63.1)	
	Trend.test	1629	951 (58.4)	
60−69				
	ALI_Tertile1	582	316 (54.3)	
	ALI_Tertile2	558	324 (58.1)	
	ALI_Tertile3	626	385 (61.5)	
	Trend.test	1766	1025 (58)	
70 or more				
	ALI_Tertile1	708	318 (44.9)	
	ALI_Tertile2	417	214 (51.3)	
	ALI_Tertile3	296	161 (54.4)	
	Trend.test	1421	693 (48.8)	

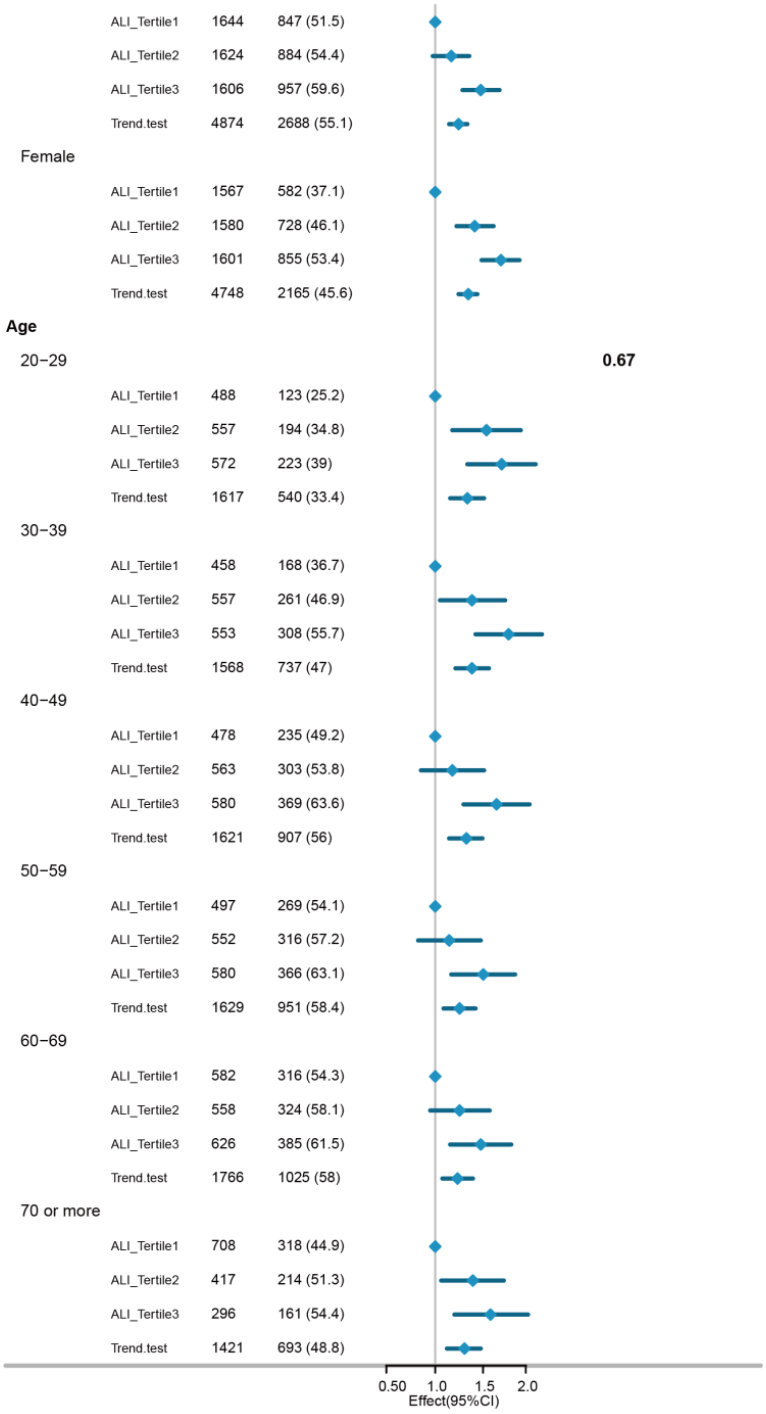

Supplementary Tables: Uploaded in the Appendix file (xls))
